# Non-Wettable Galvanic Coatings for Metal Protection: Insights from Nature-Inspired Solutions

**DOI:** 10.3390/ma18122890

**Published:** 2025-06-18

**Authors:** Ewa Rudnik

**Affiliations:** Faculty on Non-Ferrous Metals, AGH University of Krakow, Al. Mickiewicza 30, 30-059 Krakow, Poland; erudnik@agh.edu.pl

**Keywords:** superhydrophobicity, superoleophobicity, superamphiphobicity, slippery coatings, electrodeposition, surface modification, corrosion resistance

## Abstract

Natural surfaces, such as lotus leaves, springtail cuticles, and pitcher plant peristomes, exhibit extraordinary wetting behaviors due to their unique surface topographies and chemical compositions. These natural architectures have inspired the development of wettability models and the production of artificial surfaces with tailored wettability for advanced applications. Electrodeposited metallic coatings can imitate the wettability behaviors of natural surfaces, showing superhydrophobic, superoleophobic, or slippery characteristics. Such coatings can significantly enhance corrosion resistance by minimizing water–metal contact and promoting self-cleaning effects. This review presents various strategies for fabricating corrosion-resistant metallic coatings, including different electrodeposition techniques in aqueous or non-aqueous baths, followed by post-treatment procedures and surface functionalization methods. However, despite the promising protective properties demonstrated under controlled laboratory conditions, long-term studies under natural exposure conditions are still lacking, which limits the full assessment of the durability and effectiveness of non-wettable electroplated deposits in practical applications.

## 1. Introduction

Corrosion of metallic structures involves their gradual degradation caused by physicochemical interactions between the material and surrounding environment, which can ultimately lead to complete loss of functionality in a technical system [1]. This phenomenon affects key sectors of the economy, such as transportation, energy, construction, agriculture, and industry, where metallic components play a crucial role in ensuring operational continuity and structural integrity. According to a report published by NACE International in 2016 [2], the annual global cost of corrosion amounts to 3.4% of the world’s gross domestic product, while in individual countries, it can reach 1–5% of their gross national product [2,3,4,5]. These high costs arise not only from direct expenses related to maintenance, inspection, and replacement of corroded elements but also from indirect harms caused by unplanned downtime, reduced efficiency, and system failures. Critical infrastructure, such as pipelines, bridges, offshore platforms, ships, aircraft, and industrial facilities, is particularly susceptible to corrosion-related degradation. Failures in such systems may lead to significant financial losses and, more importantly, pose serious safety risks, including serious accidents [4,6]. To mitigate these risks and extend the service life of metallic components, various corrosion protection strategies are employed. These comprise material selection, design optimization, environmental control, cathodic protection, protective coatings, and the use of corrosion inhibitors [1].

The application of coatings (polymer-based, metal, or conversion layers) remains one of the most widely used and effective methods. Such coatings act as physical barriers or provide electrochemical protection, significantly reducing the rate of substrate material degradation. In recent years, advanced coatings with superhydrophobic and/or superoleophobic properties have attracted considerable interest due to their ability to form robust, self-cleaning barriers that effectively avoid the penetration of corrosive agents to metal surfaces [7,8,9,10,11,12]. The fabrication of such non-wettable surfaces depends on the choice of coating material and can be achieved through a variety of techniques, including top-down approaches (e.g., etching, anodization, laser texturing, templating) and bottom-up methods (e.g., sol-gel processing, layer-by-layer assembly, electrospinning, physical vapor deposition, chemical vapor deposition, electrodeposition, hydrothermal synthesis) [13]. The use of polymeric materials, such as fluorinated or silicone-based compounds, is the most straightforward, as they inherently possess low surface energy and can be easily structured on the micro- and nanoscale. Nevertheless, their poor adhesion to metal substrates and limited resistance to thermal and chemical stresses significantly hinder their long-term performance in aggressive environments and under frictional wear [7,13,14]. By contrast, metallic coatings offer superior mechanical strength, excellent adhesion, and enhanced stability under extreme conditions, making them a more reliable choice for corrosion protection in industrial applications [1,8,9,10,11,12].

This paper aims to review strategies for developing innovative liquid-repellent and slippery metallic coatings produced by electrodeposition, with particular emphasis on their unique surface architecture, electrochemical fabrication techniques, and chemical modifications that impart distinctive anticorrosive properties. Electrochemical methods offer accurate control over coating thickness, composition, and surface morphology, enabling the formation of functional layers even on substrates of complex geometries. Inspired by naturally non-wettable surfaces that exhibit exceptional resistance to water and/or oils, these advanced materials can imitate nature’s solutions to control liquid behavior. Through a biomimetic approach, electrodeposited coatings can achieve improved durability and self-cleaning performance, making them highly effective in protecting metal substrates under different environmental conditions.

## 2. Materials and Methods

This review is based on a comprehensive literature search focusing on publications relevant to the development of superhydrophobic, superoleophobic, superamphiphobic (an explanation of the terminology is provided in the Appendix A), and slippery metallic coatings obtained by electrochemical methods. The search strategy involved the use of targeted keywords related to surface wettability, electrodeposition of coatings, surface modification, and anticorrosion performance. Sources were selected for in-depth analysis, including journal papers, scientific books, and freely accessible institutional reports. The primary databases explored were Web of Science and Scopus, supplemented by searches in major scientific publishers’ platforms, such as the American Chemical Society, IOPscience, Royal Society of Chemistry, Taylor & Francis Online, ScienceDirect, SpringerLink, and Wiley Online Library. References were selected based on careful examination of abstracts and full texts. Particular emphasis was placed on the innovative approaches, novelty of results, and their applicability to the current review objectives. The collected literature served as the basis for the analysis and discussion presented in the following sections of this paper.

## 3. Non-Wettable Surfaces

Liquids naturally spread over solid surfaces, wetting them to a degree determined by the surfaces’ physicochemical properties. Depending on these characteristics, various wetting behaviors can be observed, including superhydrophobic surfaces that repel water [15], superoleophobic surfaces that inhibit the spreading of oils [16], and superamphiphobic surfaces that resist both water and oils [17]. Such surfaces are characterized by the ability of a liquid droplet to bead up (i.e., a contact angle greater than 150°) and to roll off easily, indicating low contact angle hysteresis (typically below 5–10°). Studies of natural surfaces [18,19,20], as well as fundamental models (Table 1), have demonstrated that two key features are essential for achieving super-repellent behavior in technical materials: a specific surface topography and low-surface-energy chemistry. Both factors contribute to the entrapment of air at the solid–liquid interface, significantly reducing attractive interactions between the surface and the liquid.

The wettability of rough surfaces by liquids (mainly water) is commonly described by two principal models, both derived from the fundamental Young equation, which applies to ideal smooth and homogeneous surfaces [21]. The Wenzel model [22] is related to homogeneous wetting, where a liquid droplet remains in contact with both the peaks and valleys of an irregular surface. In this case, increased surface roughness enhances the solid–liquid interfacial area, causing the droplet to become pinned and inhibiting its ability to roll off easily. In contrast, the Cassie–Baxter model [23] defines heterogeneous wetting, where the droplet rests on the surface asperities while air remains trapped in the underlying cavities. Greater roughness promotes the formation of air pockets beneath the droplet, thereby reducing adhesion and allowing liquid to roll off more readily. The two models predict different wetting behaviors, with the Cassie–Baxter state typically exhibiting higher apparent contact angles than the Wenzel state. However, if the air pockets beneath the droplet collapse due to liquid infiltration, an irreversible transition from the Cassie–Baxter to the Wenzel state may occur [27]. Such a transition can be induced by various factors, including chemical or topographical heterogeneity of the surface, although contact time, rather than contact area, can play a more significant role in interpreting water contact angle behavior. In turn, Marmur [23,28] discussed the Wenzel and Cassie–Baxter models from a thermodynamic perspective, introducing a new condition for heterogeneous wetting and showing that its violation leads to homogeneous wetting, even if the Cassie–Baxter equation holds. The stable hydrophobic behavior of rough surfaces is, however, more closely associated with hierarchical micro/nanoscale dual-tier structures than with surfaces featuring only microscale roughness. Verho et al. [25] reported a reversible and spatially localized pressure-induced transition between two distinct Cassie-type wetting states, arising from the two hierarchical levels of surface topography. It was shown that in the presence of such hierarchical roughness, nanoscale structures can inhibit the transition from a micro-Cassie state to a Wenzel state by maintaining trapped air pockets within the nanoscale gaps, thereby allowing a shift only to a nano-Cassie state.

**Figure 1:**
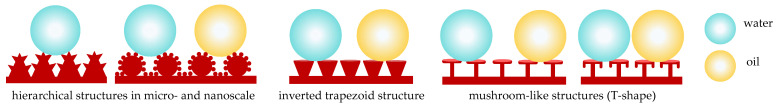
Exemplary schematic topographies of non-wettable solid surfaces.

Surface topography plays a crucial role in achieving a superhydrophobic effect, particularly through the use of hierarchical structures that combine micro- and nanoscale features. These structures facilitate the entrapment of air within surface irregularities, significantly reducing the actual contact area between the liquid and the solid, thereby increasing the apparent contact angle. Surface superoleophobicity is more difficult to achieve due to the lower surface tension of oils and other nonpolar organic liquids (usually 20–40 mN/m) compared to water (73 mN/m). In such cases, re-entrant structures with overhanging, inverted trapezoid, hoodoo-like, or mushroom-like profiles are essential, as they more effectively trap air pockets within surface irregularities and prevent liquids from penetrating into surface valleys [29,30]. These configurations ensure that contact occurs only at the surface peaks, effectively resisting infiltration by low-surface-tension liquids. To further enhance superoleophobicity, the surface energy of the surface material must also be extremely low, typically on the order of a few millinewtons per meter, like for fluorinated compounds. This combination of surface chemistry and topography (Figure 1) is critical for repelling both water and oils and achieving stable superamphiphobic behavior [15,16,29,30].

An alternative strategy for creating omniphobic surfaces involves the use of slippery liquid-infused porous surfaces (SLIPS) [26]. These systems employ a lubricating liquid retained within a micro/nanostructured matrix to form a smooth and highly repellent interface. Unlike traditional superhydrophobic and superoleophobic surfaces, SLIPS can repel a wide range of liquids regardless of their surface tension, while also providing excellent stability and self-healing capabilities.

## 4. Superhydrophobic Coatings

### 4.1. Nature Inspirations

Water repellency is not uncommon among biological surfaces in nature [18,31,32,33,34,35,36,37,38,39,40,41,42,43,44,45,46,47,48]. This phenomenon has been the subject of intensive investigation since the 1970s [49], highlighting the role of microstructural diversity in the outer surfaces of plants and animals in wettability by water (Table 2). A major breakthrough came in the late 1990s, when Neinhuis and Barthlott [31] demonstrated a relationship among surface hierarchical roughness, wettability, and the removal of contaminants from the upper surface of lotus leaves already at very low tilt angle (about 3°). Their findings provided a fundamental explanation for the self-cleaning properties of certain plant surfaces, called the ‘lotus effect.’ This discovery prompted further exploration of diverse wetting behaviors in nature, leading to the identification of other superhydrophobic modes such as the ‘rose-petal effect’ [37], where high water contact angles (WCAs) coexist with strong droplet adhesion, and the ‘Salvinia paradox’ [50], characterized by stable air retention on underwater surfaces.

**Figure 2:**
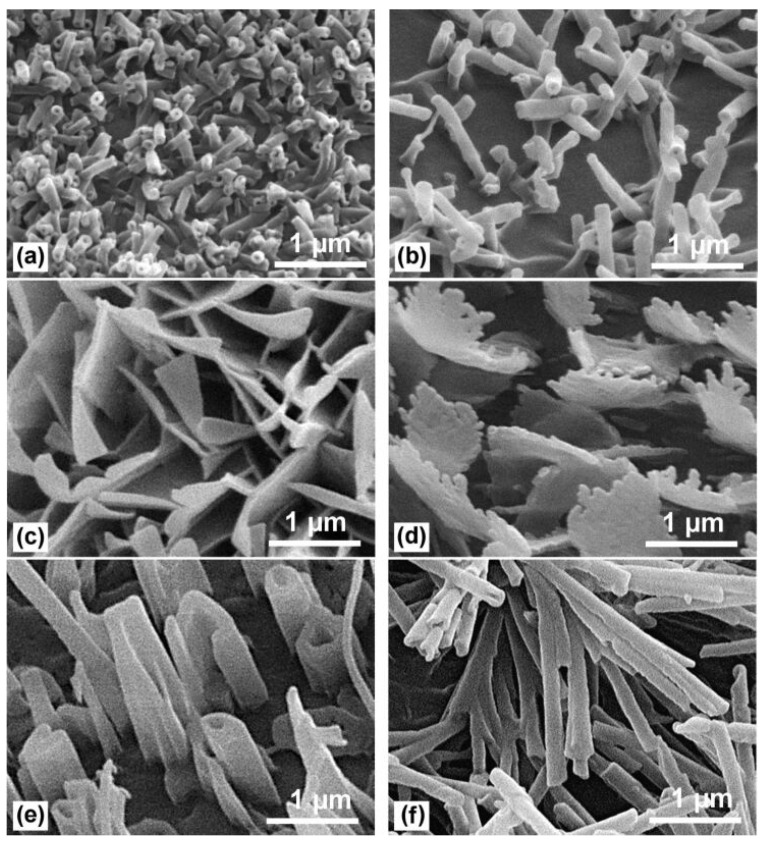
Epicuticular wax crystals on leave surfaces: (**a**) lotus—upper side, (**b**) lotus—underside, (**c**) myrtle spurge *Euphorbia myrsinites*, (**d**) Adam’s needle *Yucca filamentosa*, (**e**) wild cabbage *Brassica oleracea*, and (**f**) mottlecah *Eucalyptus macrocarpa*. Reproduced from [33] under License CC BY 2.0.

The superhydrophobicity of natural surfaces arises from two main types of surface morphology: binary micro- and nanostructures and unitary micro-line structures [34]. Hierarchical surface topographies typically consist of uniformly distributed microscale protrusions and valleys decorated with nanoscale hydrophobic epicuticular wax crystals of various shapes (Figure 2). The microscale hills and valleys minimize the contact area with water, while the nanostructured waxes prevent water penetration into the valleys. This synergistic effect leads to the formation of nearly spherical water droplets that readily roll off the surface at very low tilt angles, as observed on lotus and nasturtium leaves. In certain cases, such as rice leaves [32,34] and butterfly wings (e.g., *Morpho*, *Papilio*) [39,40], binary structures are arranged in a one-dimensional, parallel manner, producing anisotropic wettability that allows droplet movement in only one direction. Notably, in many plants (e.g., lotus, canna, and rice leaves), only the upper leaf surface exhibits superhydrophobic properties due to the presence of hierarchical structures, while the lower surface differs significantly. In contrast, other plants, such as purple setcreasea (WCAs of 167°/165°) and perfoliate knotweed (WCAs of 162°/163°), exhibit similar hierarchical structuring on both leaf surfaces, resulting in overall superhydrophobicity [34].

Unitary superhydrophobic surface structures, which are less common in nature, consist of uniformly distributed networks of slick microfibers that can trap air and minimize contact with water droplets [34,46]. Such structures have been observed on the leaf undersides of ramee *Boehmeria nivea* (WCA 164°) [34], *Hamistepta lyrata* (WCA 153°) [46], and *Artemisia umbrosa* (WCA 152°) [46], as well as on the surface of Chinese watermelon fruit (WCA 159°) [34]. Additionally, hairy leaf surfaces, such as those of lady’s mantle *Alchemilla vulgaris* L., exhibit efficient water repellency. When a droplet is deposited on such surfaces, it deforms the surface hairs, but their rigidity prevents the droplet from making contact with the substrate, allowing it to remain suspended (fakir state) [47].

**Figure 3:**
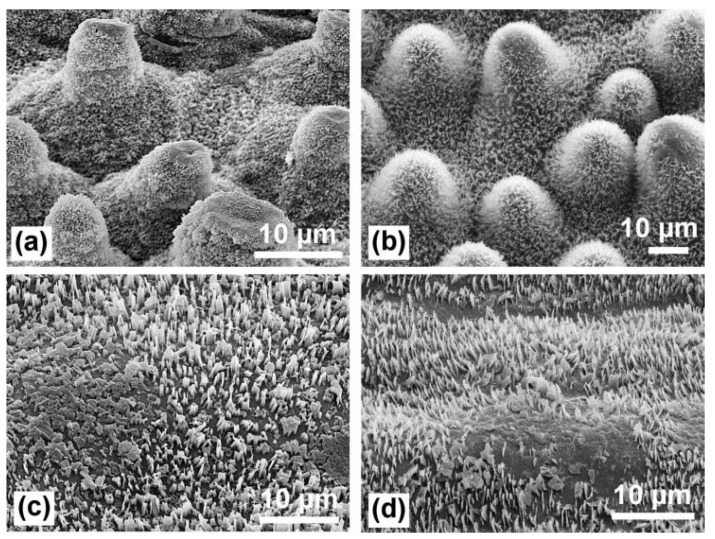
Traces of natural erosion of wax crystals on leaf surface: (**a**) lotus *Nelumbo nucifera*—upper side, (**b**) myrtle spurge *Euphorbia myrsinites*, (**c**) wild cabbage *Brassica oleracea*, and (**d**) Adam’s needle *Yucca filamentosa*. On the papillose leaves (**a**,**b**), the eroded areas are limited to the tips of the papillae, while the damaged areas can be much larger on non-papillose cells. Reproduced from [33] under License CC BY 2.0.

The superhydrophobic properties of some natural surfaces can gradually diminish over time, shifting towards higher wetting behavior [32,36,39,52]. For instance, Han et al. [39] observed that the underside of *Papilio ulysses* butterfly wings exhibited stronger hydrophobicity under continuous water contact, while the upper side showed reduced superhydrophobicity after prolonged submersion. This change was attributed to the absence of fine hairs on the upper side of the wings, which were present on the underside between the scales. Water repellency in plants can also change throughout the lifespan of their leaves [32,52]. The waxes covering superhydrophobic leaves are relatively soft and can be damaged or eroded (Figure 3), leading to stronger pinning of water [32]. However, the robustness of leaf papillae, along with their high density and massivity, helps protect the leaf against mechanical damage [33]. Gou and Guo [52] compared the wettability of clover and lotus leaves, which both exhibit similar water-repellent properties at different growth stages (tender, mature, senescent). They found that the wettability of lotus leaves increased at the senescent stage (WCA 135°), while the wettability of clover leaves remained relatively stable (WCA 140–145°) due to the enhanced mechanical stability of the epidermal tissues in the latter. Gradual drying of leaves can also lead to the degradation of micro- and nanostructures and the formation of hydrophilic chemical compositions, as observed for rape leaves [36].

Natural superhydrophobic surfaces provide valuable insights for the development of synthetic materials, as their durability is inherently influenced by factors such as mechanical wear and environmental degradation. These observations are crucial for optimizing metal coatings and ensuring their robustness and long-term performance in practical applications not only for anti-corrosion but also for anti-icing, anti-fouling, and anti-bacterial purposes.

### 4.2. Electrodeposited Coatings

The surface morphology of electrodeposited metal layers is influenced by numerous factors. These include not only the applied current or potential but also the specific composition of the electrolyte bath, such as the presence of particular anions and inorganic or organic additives. A distinct hierarchical surface structure can be achieved primarily through the use of various electrolysis modes in aqueous solutions, often followed by chemical surface modification with low-surface-energy compounds or thermal (oxidation) treatment to impart highly hydrophobic properties. Alternatively, electrodeposition in non-aqueous electrolytes may offer a single-step approach to achieve the required surface characteristics. Table 3 and Table 4 show exemplary cases that illustrate the variety of fabrication conditions and the corresponding properties of the obtained superhydrophobic metal coatings.

#### 4.2.1. One-Step Electrolysis

One-step electrolysis conducted under galvanostatic or potentiostatic conditions represents the simplest approach to coating deposition. However, achieving surface hydrophobicity using conventional aqueous electrolytes remains challenging, even though chloride anions enhance surface roughness and sulfate anions exhibit a leveling effect [53]. Moreover, the wettability of the coating depends on the applied current parameters, and for each set of conditions, there are optimal current density, potential, and electrolysis time values that ensure the desired surface properties [53,54,55,56,57,58,59,60,61,62,63,64,65,66,67,68,69,70,71,72,73,74,75,76,77,78,79,80,81,82,83,84]. A natural increase in hydrophobicity is sometimes observed during sample storage, which may be attributed to the spontaneous formation of an oxide layer on the coating surface or the adsorption of organic compounds from the air [55,56]. Notably, the adsorption of trace atmospheric hydrocarbons, combined with spontaneously formed oxide layers, can contribute to corrosion protection by minimizing water contact and sustaining long-term superhydrophobic stability.

**Table 3:** Fabrication techniques and properties of superhydrophobic coatings electrodeposited from aqueous electrolytes.

Hydrophobicity Drivers	Coating	Fabrication Technique	Surface Topography	WCA	Corrosion Resistance *	Ref.
**One-step Electrolysis**						
surface roughness	Ni	chloride bath + C_2_H_10_Cl_2_N_2_ modifier galvanostatic deposition	hierarchical	150–155°	−220 mV 10^−9^–10^−8^ A/cm^2^	[54]
	Ni	sulfate–ethanol bath + C_12_H_24_O_2_ modifier, galvanostatic deposition	hierarchical micro-flower	168°	−550 mV 10^−6^ A/cm^2^	[57]
	Cu-Mn	chloride bath + C_14_H_28_O_2_ modifier, galvanostatic deposition	hierarchical masonry-like	161°	−450 mV 10^−5^ A/cm^2^	[58]
	Zn-Ni	chloride–sulfate–gluconate bath, galvanostatic deposition, hydrogen-bubble template	porous cauliflower-like clusters	152°	−1360 mV 10^−7^ A/cm^2^	[59]
molecules of low surface energy	Zn	acetate bath, potentiostatic deposition; modification with stearic acid	multiscale needle and branch-shaped fractal	156–166°	−960 mV 10^−5^ A/cm^2^	[60]
	Ni	chloride bath, galvanostatic deposition; electrochemical modification with polysiloxane	microcones	157°	−270 mV * 10^−8^ A/cm^2^	[64]
	Ni	chloride bath, galvanostatic deposition; modification with myristic acid	hierarchical starfishes	152–157°	−482 mV 10^−8^ A/cm^2^	[65]
	Ni-Fe	chloride–sulfate–glycerol bath, galvanostatic deposition; modification with myristic acid	hierarchical *Echinopsis multiplex* (cactus)-like	166°	−838 mV 10^−6^ A/cm^2^	[68]
oxide layer	Sn	chloride bath, potentiostatic deposition; post-annealing in air	porous tremella-like	170°	one-year stability in air	[72]
	Zn	acetate bath, potentiostatic deposition; post-annealing in air	willow-leaf-like	170°	−240 mV 10^−10^ A/cm^2^	[73]
**Two-step Electrolysis**						
surface roughness	Ni	chloride bath + C_2_H_10_Cl_2_N_2_ modifier; galvanostatic depositions	micro- and nanocones	156°	−140 mV 10^−6^ A/cm^2^	[77]
	Ni-Zn	chloride bath + NH_4_Cl modifier; galvanostatic depositions	micro- and nanocones	155°	−245 mV 10^−6^ A/cm^2^	[78]
molecules of low surface energy	Cu	sulfate bath, potentiostatic depositions; modification with stearic acid	hierarchical cauliflower-like	160–164°	−220 mV 10^−9^–10^−6^ A/cm^2^	[63]
**Electrochemical Additive Manufacturing**						
surface roughness	Ni	chloride bath + NH_4_Cl modifier; galvanostatic scanning deposition	porous cauliflower-like clusters	155°	stable properties in water (after 6 months) or hot air	[81]
	Ni	chloride–sulfate bath + Ni nanoparticles; galvanostatic scanning deposition + magnetic field	porous cauliflower-like clusters	155°	−240 mV 10^−8^ A/cm^2^	[82]
molecules of low surface energy	Cu	sulfate bath; galvanostatic jet deposition; modification with stearic acid	hierarchical cauliflower-like	151°	−225 mV 10^−5^ A/cm^2^	[83]

* Corrosion potential versus reference electrode (saturated calomel electrode or Ag/AgCl).

Various bath additives are typically employed to influence crystallite growth, leading to changes in surface morphology. Farzaneh et al. [54] demonstrated that nickel coatings deposited from a chloride bath with ethylenediamine dihydrochloride C_2_H_10_Cl_2_N_2_ can achieve superhydrophobicity when optimized for current density and electrolysis time, forming micro/nanobinary structures and significantly improving corrosion resistance. Compared to hydrophilic nickel coatings, the corrosion current density decreased by 2–3 orders of magnitude, and the corrosion potential shifted by approximately +20 mV. Zhou et al. [57] achieved similar effects using a water–ethanol sulfate bath modified with lauric acid C_12_H_24_O_2_, obtaining coatings with a water contact angle near 170°, excellent durability, and self-cleaning properties stable up to 7 days. Tang et al. [58], in turn, used myristic acid (typically used as a post-treatment agent) as a bath component for Cu–Mn codeposition, producing multilayer micro/nanostructured coatings with a contact angle of 160° and high stability under mechanical, thermal, and chemical stress. In turn, Wang et al. [84] used choline chloride as a nickel crystal modifier to produce self-cleaning corrosion-resistant coatings with self-recovery superhydrophobicity properties (WCA 160–162°) during air exposure (3 days).

An unconventional electrodeposition method for superhydrophobic Zn–Ni alloy coatings was applied by Wang et al. [59]. They produced porous layers using a dynamic hydrogen template, achieving the development of various structures with micropores and polyhedral grains forming rose- or cauliflower-like clusters. The size of the micropores decreased with intensified hydrogen evolution caused by increased temperature (up to 50 °C). The as-prepared superhydrophobic coatings exhibited a mirror-like effect (a silvery appearance of the sample immersed in water due to a superficial air layer), stability under water jet impact, excellent self-cleaning properties, and anticorrosion performance (corrosion protection efficiency decreased by about 15% after 10 h immersion in sodium chloride NaCl solution).

Electrodeposition, followed by surface modification, is the most common approach to fabricating superhydrophobic coatings. Chemical modification is typically performed either by immersing the substrate in an alcohol solution (methanol, ethanol) of a functionalizing agent for a certain period [60,61,62,63], by spraying the compound onto the surface [66], or by using electrochemical methods [64,67]. It has been reported [7] that surface energy in monolayer films decreases in the order –CH_2_ > –CH_3_ > –CF_2_ > –CF_2_H > –CF_3_; thus, long-chain carboxylic acids (e.g., stearic acid [60,61], myristic acid [65,67,68]), polymers (e.g., silicones [64,66], fluoropolymers [63,69]), and n-dodecanethiol [70,71] are among the most commonly used compounds for reducing the surface energy of metallic materials. Notably, fluorinated polyhedral oligomeric silsesquioxanes (POSS) exhibit the lowest surface energy (9 mJ/m^2^) recorded for any crystalline solid [7].

As an alternative surface modification approach, metal oxides are widely used to create superhydrophobic surfaces due to their controllable surface roughness and strong water repellence. This strategy often involves thermal post-treatment of electrodeposited coatings to form metal oxide layers [72,73]. Notably, functional coatings with new surface structures can also be fabricated directly on metal substrates through hydrothermal methods [74], coating anodization [75], or one-step electrodeposition from dilute salt solutions [76]. In the latter case, the dominant hydrogen evolution reaction promotes local pH increase near the electrode surface, inducing the hydrolysis of metal salts and leading to the in situ formation of metal hydroxides, which subsequently convert into metal oxides.

#### 4.2.2. Two-Step Electrolysis

Two-step electrolysis conducted under galvanostatic or potentiostatic conditions is a common approach for tailoring surface morphology [62,77,78,79,80]. In this method, a low current density (or slightly negative potential) applied for a prolonged time promotes the formation of uniform microstructures, while a subsequent short pulse of high current density (or strongly negative potential) induces the development of nanoscale features, resulting in hierarchical micro/nanostructured surfaces. It is important to note that the goal of this process is to create a superficial morphology that is robust and facilitates the trapping of air bubbles in surface valleys, but not to form dendritic coatings with weak adhesion. For example, electrodeposition of nickel [77,79] or nickel-rich alloys [78] from chloride baths containing crystal modifiers such as ethylenediamine dihydrochloride C_2_H_10_Cl_2_N_2_ [77,79,80] or ammonium chloride NH_4_Cl [78] of high concentrations led to the formation of hierarchical micro- and nanocone surface topographies. This mode of formation and growth of Ni micro–nanocones was attributed to the tip-discharge effect [79]. Initially, the nickel coatings exhibited superhydrophilicity (WCA ~5°), but after two weeks of air storage, their wettability significantly decreased, transforming the surfaces into superhydrophobic ones (WCA 156°) [77]. The issue of initially weak hydrophobicity is not observed in nickel–zinc alloys with similar surface structures, which exhibit superhydrophobic properties immediately after deposition [78].

Two-step electrodeposited coatings can also be functionalized through chemical modification. Mousavi and Pitchumani [62] deposited copper at constant potentials, followed by surface treatment with stearic acid. The resulting coatings were tested for corrosion resistance in NaCl solutions across a wide pH range (1, 7, and 14), demonstrating protection efficiencies of up to 99% after 72 h. Consistently stable superhydrophobicity (WCA 153–161°) was also maintained in solutions with varying pH (1–14) or salinity (1–5 M).

#### 4.2.3. Electrochemical Additive Manufacturing

Electrochemical additive manufacturing has attracted intensive attention in recent years due to the possibility of fabricating complex three-dimensional micro- and nanostructures with controlled porosity, finer grain sizes, and faster deposition rates [85]. Scanning jet electrodeposition enables localized coating growth by synchronizing deposition with the movement of a precisely controlled anode nozzle. This technique offers high flexibility and portability, as both the nozzle’s shape and outlet size can be tailored to specific requirements. Compared to traditional electrodeposition, it provides greater efficiency due to the continuous delivery of fresh electrolytes to the cathode surface, which minimizes concentration polarization and allows for the use of higher current densities. Shen et al. [81] applied this novel technique to fabricate nickel coatings, which were initially porous and hydrophilic but became superhydrophobic after one week of air exposure, exhibiting a sliding angle of about 6°. This transformation was attributed to the adsorption of airborne hydrocarbons. The method was later developed [82] by introducing spherical nickel nanoparticles into the plating bath and applying a magnetic field. At an optimal magnetic field strength of 120 mT, ferromagnetic particles were incorporated into the growing nickel layer, forming a hierarchical structure that developed superhydrophobic properties (after five days of air storage), along with improved corrosion resistance. In turn, Jinsong et al. [83] applied jet electrodeposition to prepare copper coatings. The as-plated surface was hydrophobic (WCA 145°), which became superhydrophobic (WCA 152°) after stearic acid modification. After 144 h of immersion in NaCl solution, the coating’s corrosion current density was reduced by an order of magnitude compared to pure copper, indicating enhanced corrosion resistance. Additionally, after one month of exposure to a damp atmosphere, the water contact angle decreased by only 2°, demonstrating good stability of surface properties.

#### 4.2.4. Non-Aqueous Electrolytes

Non-aqueous electrolytes, including conventional organic solvents, deep eutectic solvents, and ionic liquids [86,87,88], offer promising alternatives to aqueous baths for the electrodeposition of metals and alloys. While aqueous electrolytes provide mild operating conditions and good solubility for metal salts, they are often limited by hydrogen coevolution and hydrogen embrittlement, which reduce current efficiency and coating quality. In contrast, non-aqueous systems expand the range of metals that can be deposited, enable better control of deposition parameters, and allow electrodeposition under wider electrochemical windows. Moreover, they facilitate the formation of distinct surface morphologies combined with the presence of superficial organic hydrophobic compounds, which can be tailored for specific functional properties like low wettability and improved corrosion protection [89,90,91,92,93,94,95].

**Table 4:** Fabrication techniques and properties of superhydrophobic coatings electrodeposited from non-aqueous electrolytes.

Hydrophobicity Drivers	Coating	Fabrication Technique	Surface Topography	WCA	Corrosion Resistance *	Ref.
**Conventional Solvent-based Baths**						
surface roughness of low surface energy	Ni	chloride–ethanol bath + myristic acid modifier; one-step galvanostatic deposition	hierarchical protrusions	172°	−166 mV 10^−9^ A/cm^2^	[80]
	Mn	chloride-DMSO bath + stearic acid modifier; one-step galvanostatic deposition	porous cauliflower-like clusters	154–159°	10^6^ Ω∙cm^2^	[90]
**Deep Eutectic Solvent-based Baths**						
surface roughness of low surface energy	Zn	chloride–choline chloride–ethylene glycol bath + stearic acid modifier; one-step galvanostatic deposition	hierarchical ordered micro-slices and nanoconcaves	165°	−920 mV 10^−6^ A/cm^2^	[91]
	Ni	chloride–choline chloride–ethylene glycol bath + stearic acid modifier; constant voltage deposition	hierarchical flowers, nanostrips or nanosheets	162–166°	−710 mV 10^−6^ A/cm^2^	[92]
	Cu	chloride–choline chloride–ethylene glycol bath + stearic acid modifier; one-step galvanostatic deposition	porous layered clusters	158°	−180 mV 10^−7^ A/cm^2^	[93]
molecules of low surface energy	Cu	chloride–choline chloride– ethylene glycol bath; one-step galvanostatic deposition; modification with stearic acid	porous broccoli-like clusters	152°	−230 mV 10^−7^ A/cm^2^	[94]
	Cu	chloride–choline chloride– ethylene glycol bath; one-step galvanostatic deposition; modification with stearic acid	flower-like clusters	158°	−220 mV 10^−7^ A/cm^2^	[95]
	Zn	chloride–choline chloride–ethylene glycol bath + thiourea modifier; one-step galvanostatic deposition; modification with polypropylene	spongy microscale network structure with tiny sheets	170°	−934 mV 10^−5^ A/cm^2^	[96]

* Corrosion potential versus reference electrode (saturated calomel electrode or Ag/AgCl).

Conventional non-aqueous electrolytes using anhydrous ethanol [80,89,90] or dimethyl sulfone (DMSO) [90] as solvents offer cost-effective baths, as they enable the dissolution of higher carboxylic acids conventionally used as post-electrodeposition modifiers in coatings deposited from typical aqueous baths. Daneshnia et al. [80] compared the characteristics of nickel coatings obtained using a two-step electrolysis process from an aqueous chloride bath and a one-step electrolysis from a chloride ethanolic bath containing myristic acid. In both cases, hierarchical surface structures were formed; however, while the aqueous bath produced micro- and nanocones [77], the non-aqueous bath resulted in spherical surface features. Despite the lower roughness of the latter, superhydrophobicity was achieved without the need for air storage, and the coating also demonstrated improved long-term corrosion resistance (after 9 days of immersion in saline solution) due to the presence of superficial nickel myristate [89]. In turn, Zaffora et al. [90] deposited manganese coatings from electrolytes containing the same concentrations of metal salt and stearic acid modifier, but using different solvents like ethanol and DMSO. As deposition time increased (20–60 s), the hydrophobicity of the coatings improved; however, superhydrophobicity was achieved only with the DMSO-based solution. This difference was attributed to the resulting surface morphology: Ethanol produced surfaces covered with nanoneedles that aggregated into larger structures without fully covering the substrate, whereas DMSO led to a uniformly distributed porous hierarchical topography. Additionally, the presence of manganese stearate contributed to enhanced non-wettability, imparted self-cleaning properties, and significantly improved corrosion resistance by an order of magnitude compared to the steel substrate.

Deep eutectic solvents are formed by mixing two or more molecular components into a eutectic system with a melting point lower than that of the individual substances [88]. Typically, they consist of choline chloride with ethylene glycol (1:2) and a hydrated metal salt (primarily chlorides). Such electrolytes offer several attractive properties, including good thermal and chemical stability, high solubilizing capacity for metal salts and higher carboxylic acids, and low vapor pressure. These characteristics, combined with their simple and low-cost preparation, make deep eutectic solvents significantly more accessible for the electrodeposition of superhydrophobic coatings than conventional non-aqueous systems. Interestingly, using choline chloride–ethylene glycol solvents with stearic acid as an additive allows the formation of different superhydrophobic surface types. For example, Li et al. [91] produced zinc coatings exhibiting the rose petal effect, while Gu and Tu [92] obtained nickel coatings showing the lotus effect. In both cases, superhydrophobicity resulted from the formation of metal stearate structures on the surface, although different current modes were applied, i.e., constant current [91] and constant or pulsed voltage [92]. Alternatively, deposits produced in deep eutectic solvents can be modified with stearic acid in a subsequent step. It has been shown [94,95] that copper coatings can exhibit different super-non-wettability properties depending on the modification duration. Shorter immersion times are more favorable due to the formation of less porous stearic acid layers, which lead to higher water contact angles while maintaining corrosion resistance at comparable levels (one order higher than non-treated deposits [93,94,95]) and self-cleaning properties. Noteworthy, post-treatment of zinc coatings with a nonpolar polymer (polypropylene dissolved in dimethylbenzene) yielded superior results, as the cured polymer formed a thin film with excellent non-wettability (WCA 170°), effectively providing a physical barrier that isolated the substrate from the corrosive environment [96].

Ionic liquids are composed of relatively large organic cations with small organic or inorganic anions [86,87]. Their properties can be designed by modifying the ion structure, though this often requires complex synthesis, making them relatively costly compounds. Although ionic liquids are known for their wide thermal and chemical stability and high conductivity and broad electrochemical stability windows, there is a lack of available information on the use of this group of electrolytes for producing superhydrophobic electrodeposited metallic coatings.

## 5. Superhydrophobic-(Super)oleophobic Coatings

### 5.1. Nature Inspirations

**Figure 4:**
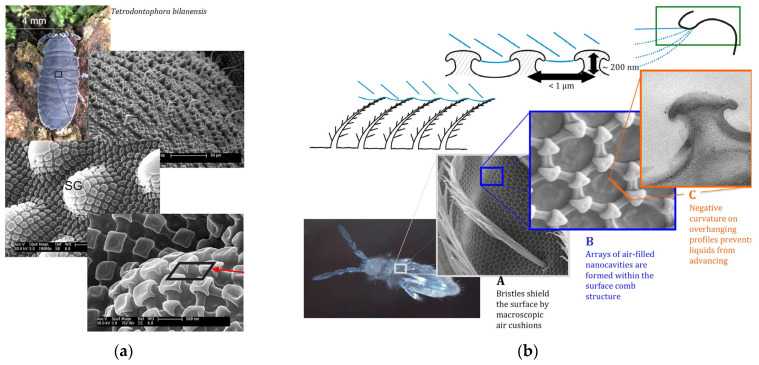
Surface topography of springtail cuticles (**a**,**b**) featuring three levels of wettability protection (**b**). Reproduced from [97] under License CC BY 4.0.

Simultaneous water and oil repellency is relatively rare in nature compared to hydrophobicity alone. While many surfaces found in flora and fauna effectively repel water, only a few have developed specialized structures and surface chemistries that provide resistance to both polar and nonpolar liquids (superominphobicity). Typical organisms exhibiting superomniphobic body surfaces include springtails (Collembola), such as *T. bielanensis*, *P. flavescens*, *A. pygmaeus*, *S. quadrispina*, etc. [19,97]. These wingless, hexapod insects, ranging in size from 0.1 mm to 9 mm, are widespread across the Earth. Their cuticle exhibits a hierarchical topography with a re-entrant texture (Figure 4). Such surface structure has a major influence on super-wettability, often manifested as a high water contact angle of 150–170° [98,99] and non-wettability toward both polar (22–26 mN/m) and nonpolar (26–27 mN/m) organic liquids [97]. Nevertheless, different Collembola species achieve effective liquid repellency through varying strategies that involve both surface topography and chemical composition [99,100].

Non-wettability is also observed on the integument of leafhoppers (e.g., *Alnetoidia alneti*, *Athysanus argentarius*, *Cicadella viridis*), whose surfaces are coated with brochosomes exhibiting re-entrant structures [101,102]. In most species, these brochosomes are spherical, honeycomb-like particles measuring 200–700 nm in diameter, with pentagonal and hexagonal wall compartments that open into a hollow core. It has been shown that on intact leafhopper wings, the average water contact angles can reach as high as 160–172°, while the contact angles of ethylene glycol (48 mN/m) and diiodomethane (50 mN/m) on brochosomal coatings were measured at 153–164° and 148–156°, respectively. Conversely, brochosomal coats were completely wettable by ethanol (22 mN/m). Thus, water repellency in brochosomal coatings can result from a rough surface texture that restricts liquid contact to asperity tops with trapped air beneath, while a low presence of polar molecules enhances repellency toward polar liquids of similar surface tension.

Bacterial biofilms provide a clear example of biological surfaces that repel liquids. These are viscoelastic materials composed of bacteria embedded within a matrix of secreted macromolecules, which protect the cells from harsh environmental conditions. Epstein et al. [103] demonstrated that *Bacillus subtilis* biofilms repel water and aqueous solutions of ethanol, isopropanol, methanol, and acetone. The observed contact angles ranged from 125° to 140°, indicating both the hydrophobic and oleophobic nature of the biofilm. Noteworthy, Werb et al. [104] observed that the wetting behavior of *Bacillus subtilis* biofilms can vary significantly, ranging from hydrophilic to superhydrophobic states characterized by either the lotus effect or rose-petal effect. These differences were associated with different surface topologies and variations in the protein composition of the bacterial colony matrix.

The characterization of non-wettable biological materials is still in its early stages, with many properties yet to be fully understood. Notably, some fish exhibit unique skin structures that are superoleophilic in air while simultaneously superoleophobic in water [105] (e.g., the underwater oil contact angle of 178° on tilapia fish scales [106]), or even show anisotropic oil droplet movement in water (e.g., on a filefish surface due to hook-like spines oriented in a single direction [107]), emphasizing the complex multifunctionality of natural liquid-repellent surfaces in air and underwater.

### 5.2. Electrodeposited Coatings

Surface microstructures of non-wettable materials are typically characterized by wetting behavior described by the Cassie–Baxter model for both high- and low-surface-tension liquids [108]. The effectiveness of liquid repellency strongly depends on surface topography, particularly on convex features that narrow downward and possess concave sidewalls, commonly referred to as re-entrant textures [29,30,108]. Such textures play a key role in enabling (super)oleophobicity by promoting stable air entrapment within the surface roughness, although such topographies alone are often insufficient. Therefore, further modification with fluorinated compounds (e.g., perfluoro octadecyltrichlorosilane, perfluorooctanoic acid, perfluorodecanethiol) of low surface tension (10–20 mN/m) is fairly common [16,17].

Electrodeposited metal coatings exhibiting (super)amphiphobic or (super)omniphobic surface properties remain relatively rare; however, interest in this area has been steadily increasing in recent years [109,110,111,112,113,114,115,116,117]. Several strategies for the fabrication of such coatings have been proposed, with aqueous electrolyte-based electrodeposition being the predominant method, while the use of deep eutectic solvents or ionic liquids has not yet been reported. One-step [109,110,111,112] or even two-step [115,116,117] electrodeposition from aqueous solutions is generally insufficient to achieve high repellence toward various liquids; therefore, this process is typically followed by chemical surface modifications, most often including simple immersion in organic solutions of perfluorooctanoic (perfluoro caprylic) acid or fluorosilanes. Notably, Qing et al. [110] applied a two-stage modification: first, treating zinc electrodeposits with a stearic acid ethanol solution to achieve superhydrophobicity, then immersing them in an ethanol-based suspension of TiO_2_ particles modified with FAS (tetrahydro perfluorodecyltrimethoxysilane) to attain superoleophobic properties. In contrast, excellent results have been achieved using non-aqueous solutions based on absolute ethanol with the addition of myristic acid, yielding contact angles of approximately 170° for water and 160° for oil [113,114].

The obtained coatings are typically evaluated for wettability using water and various liquids collectively referred to as oils. Most frequently, these include glycerol (63 mN/m) [111,113,114,115,116], unspecified oils [109,110,112,117], ethylene glycol (48 mN/m) [113,114], octane (22 mN/m) [111], and 5–25% ethanol–water mixtures (25% ethanol: 36 mN/m) [115]. Among organic compounds, superoleophobicity was achieved primarily in tests involving glycerol and oils, whereas the remaining liquids exhibited only oleophobic behavior with typical maximal oil contact angles (OCAs) of 130–140°.

Table 5 summarizes selected experimental data on superamphiphobic electrodeposited coatings. In comparison to the superhydrophobic electrodeposited coatings, it is difficult to clearly determine the influence of deposition parameters on surface wettability due to the limited number of studies. However, it appears that extending the electrolysis time, increasing the current density or voltage, and prolonging the surface modification step have a relatively minor impact on contact angle values [113,116]. More pronounced effects were observed only when metal salt concentration in the bath was changed [109] or the electrolysis mode was altered [116], thereby modifying the coating’s roughness. These coatings are primarily based on single metals, mainly zinc and nickel, which are typically applied as protective layers on steel. In contrast to conventional coatings, non-wettable layers demonstrated enhanced corrosion resistance as well as remarkable self-cleaning capabilities against various contaminants (solids, solutions, oils) due to their non-adhesive surfaces [111,112,113,114,115,116].

Additional reported properties include mirror-like effects in both aqueous and oily media [109,114], mechanical durability [112], and relative stability of (super)wettability characteristics during storage in air for several months, even though a gradual decrease in contact angle may occur over time [111,113,115,116]. These findings highlight the potential of electrodeposited coatings with amphiphobic or even omniphobic properties. Although promising repellency against a range of liquids has been achieved in laboratory conditions, the long-term stability of such coatings, especially under corrosive or operational environments, remains unexplored.

## 6. Slippery Coatings

### 6.1. Nature Inspirations

**Figure 5:**
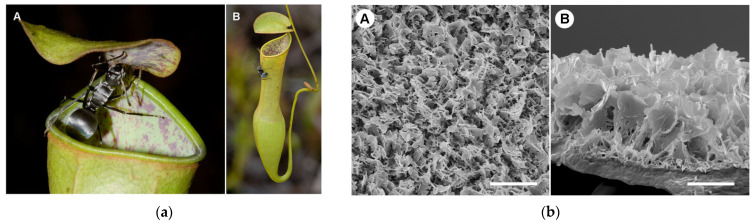
Morphology of pitcher plant *Nepenthes gracilis*. (**a**) Pitcher with visiting ant (A—epicuticular wax crystal surfaces visible on the inner pitcher wall and on the underside of the pitcher lid; B—horizontal position of the lid above the pitcher opening facilitates prey capture). (**b**) Structure of the wax crystal layer on the inner pitcher wall (A—top view; B—side view). Reproduced from [118] under License CC BY.

The most well-known example of a naturally slippery and liquid-repellent surface is the peristome of the carnivorous pitcher plant (*Nepenthes* spp.) [118]. During rainfall, the inner surface of its cupped leaf becomes extremely slick, causing insects such as ants and spiders to lose adhesion and slide uncontrollably into the trap (Figure 5a), where they are dissolved by plant digestive juices. This unique slipperiness arises from the synergistic effect of surface microstructures and a stable, thin aqueous film that acts as a lubricant. The film is retained by capillary forces and specific topographical structures (Figure 5b), effectively preventing solid–solid contact and significantly reducing friction. Inspired by this natural mechanism, a new class of self-repairing polymer materials called slippery liquid-infused porous surfaces (SLIPS) was developed in 2011 [26], exhibiting high repellency toward a wide range of liquids.

### 6.2. Electrodeposited Coatings

The electrodeposition of slippery metal coatings is still at an early stage of development, with only a limited number of examples reported to date [119,120,121,122,123,124,125,126,127]. These coatings aim to combine the mechanical durability of porous metallic structures with low-friction and liquid-repellent properties. Their performance depends on the ability of micro- and nanostructures to retain a lubricant (e.g., silicone oils, fluorocarbon oils, vegetable oils, ionic liquids [128]). The lubricant remains immiscible with dropped liquids, and due to the differences in their surface tensions, such slippery surfaces exhibit self-cleaning behavior. In fact, the liquid droplet floats on the lubricant layer instead of adhering to the substrate, enabling easy removal with minimal tilting angle (TA).

Electrodeposited slippery coatings are porous layers with various morphologies, modified with organic compounds and typically impregnated with silicone oil, perfluorinated oils, or deep eutectic solvents (Table 6). Three-dimensional metal structures can be achieved using different strategies such as (i) natural formation of dendritic crystals, as in the case of silver deposited from a nitrate bath [119], (ii) forced formation of dendritic crystals under high cathodic polarization [120,125,126], (iii) creation of porous structures via dynamic hydrogen templating [123], and (iv) two-step deposition processes with both stages conducted under potentiostatic conditions (a longer first stage at a more negative potential, followed by a shorter stage at a less negative potential) [121] or using combined potentiostatic-galvanostatic modes [122]. The deposited metals were subsequently functionalized to achieve superhydrophobicity either by conventional one-step chemical modification (e.g., exposure to dodecanethiol vapor [119], immersion in fatty acid solutions [123,127]) or through a three-step process. In the latter technique, the metal surface was oxidized chemically (in ammonium persulfate solution) [124,125] or electrochemically [124] to form an oxide layer for polydopamine anchoring, followed by grafting of long organic chains of n-dodecanethiol via vapor deposition. A dendritic copper layer was also used as a catalyst for the synthesis of carbon fibers, forming a porous layer, while the dendritic structure of copper itself disappeared during the process [126]. Alternatively, composite coatings with rough surfaces were also deposited [127].

The obtained superhydrophobic coating structures infused with liquid lubricants create slippery surfaces on which water droplets slid off quickly at low tilt angles, while superhydrophobicity was lost. Notably, Xiang et al. [123] used molten paraffin to impregnate a nickel structure, achieving stable properties during repeated heating and cooling cycles. A simultaneous self-healing effect was observed after abrasion and thermal treatment, attributed to the temperature-dependent phase transition of the lubricant between solid and liquid states. The healing property of a physically damaged surface after thermal assistance was also confirmed for other slippery coatings [124,127].

Stoddard et al. [122] performed comparative studies on the fabrication of SLIPS copper coatings (textured at different electrodeposition potentials) by testing combinations of six functionalizing agents (stearic acid, octylphosphonic acid, perfluorodecyltriethoxysilane, n-hexadecyl mercaptan, Sylgard 184, and Gentoo) with three lubricant types: perfluoropolyethers (Krytox 103, Krytox 104), silicone-based oils (Downsil 510-50 cSt and 510-100 cSt), and diphenyl ether derivative (Santolube OS-105). It was found that electrodeposited copper textures achieved superhydrophobicity (158–169°) with all functionalization agents used, although each agent exhibited different levels of durability under heated conditions (30–90 °C), static immersion (up to 7 days), and water jet impingement (10 m/s). While all agents performed well under static conditions at room temperature, only n-hexadecyl mercaptan and Sylgard 184 (a curing silicone elastomer) demonstrated significant durability under heated, static conditions and during jet impingement tests. Notably, the primary cause of property loss was water saturation within the surface asperities, whereas the morphology of the asperities and the functionalization layer itself remained unaffected. In turn, infused lubricants resulted in varying water contact angles (100–120° for mercaptan functionalizing agent) but showed nearly identical contact angle hysteresis (2–3°). Santolube OS-105 (viscosity of 109 mPa∙s) exhibited the best durability during heated water immersion, as its strong intermolecular interactions limited lubricant spreading in water, while Krytox 104 (viscosity of 100 mPa∙s) demonstrated the best durability in jet impingement tests due to its lower susceptibility to shear degradation. Under turbulent flow conditions, lubricant-infused surfaces degrade continuously and are unsuitable for harsh environments, but in milder applications such as condensate shedding, they remain durable for up to 1.5 years before requiring reapplication.

The slippery coatings demonstrated enhanced corrosion resistance not only compared to bare substrates [120,121,123,124,125,126,127] but also relative to porous structures in their superhydrophobic state [121,123,124], even in saline environments at temperatures as high as 85 °C [121]. Corrosion properties are typically assessed using rapid methods such as dynamic polarization curves [120,121,123,124,125] or electrochemical impedance spectroscopy [120,123,124,125,126]. A gradual degradation of the anticorrosive performance was often observed, indicated by an increase in corrosion current density, a shift in corrosion potential toward more negative values [121,123,124], and a rise in sliding angles with immersion time in NaCl solutions [127]. In contrast, stable corrosion resistance was reported for slippery cobalt- and iron-based coatings immersed for up to 30 days [120,125]. Notably, slippery coatings can also effectively prevent microorganism attachment (Table 7), thereby reducing the risk of biocorrosion and biofouling [119,120,124,125].

## 7. Conclusions

Nature-inspired electrodeposited metallic coatings, including superhydrophobic, superoleophobic, and slippery liquid-infused surfaces, offer a versatile base for improving the functional performance of metallic substrates. The diverse strategies used for electrodeposition and post-deposition modification of coatings reflect a high level of scientific creativity and innovation. Superhydrophobic and superoleophobic coatings, typically based on chemically modified micro/nanostructures, demonstrate excellent liquid repellency, but their durability in dynamic and corrosive environments remains a challenge. By incorporating a lubricating liquid into porous or dendritic structures, slippery coatings address some of these limitations by offering enhanced corrosion resistance, self-healing capabilities, and resistance to biofouling, particularly in saline environments and under moderate conditions.

**Figure 6:**
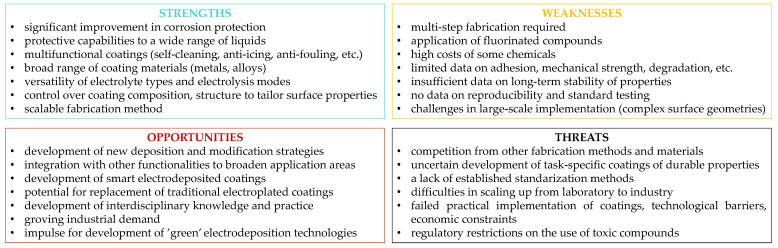
Electrodeposited non-wettable metallic coatings—SWOT analysis.

Non-wettable galvanic coatings represent a highly promising direction for corrosion protection, particularly due to their ability to trap air within surface structures and/or rapidly remove droplets from the surface, thereby effectively minimizing contact between the corrosive medium and the metal substrate. Such mechanisms are beneficial in both aqueous and non-aqueous environments. Although no single method guarantees universal protection, non-wettable coatings stand out due to their barrier-type nature, which can complement conventional corrosion inhibitors in certain applications [128]. While chemical and mechanical degradation [10,14,129] can limit the long-term performance of these coatings in harsh flow environments, they show significant potential in static and low-flow applications such as condensate management, self-cleaning, and marine antifouling systems. Their tunable properties, multifunctionality, and adaptability to various surface chemistries suggest promising opportunities for further development and implementation in targeted industrial applications (Figure 6).

The electrodeposition of non-wettable coatings represents an innovative and evolving field where biological science, material engineering, surface chemistry, and electrochemistry combine to overcome practical challenges, positioning these coatings as advanced solutions for next-generation surface technologies. Continued research should focus on improving durability under real-world exposure to natural environments and operational conditions, as well as exploring environmentally friendly fabrication methods to eliminate the need for fluorinated compounds.

## Figures and Tables

**Table 1:** Main concepts of wettability of flat and rough solid surfaces.

Model	Liquid Droplet	Contact Angle	Remarks	Ref.
Young	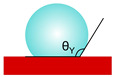	cosθ_Y_ = (γ_SG_ − γ_SL_)/γ_LG_ θ_Y_—contact angle γ—surface free energy (or surface tension)	a liquid droplet on a flat surface; an equilibrium between superficial energies at the solid–liquid–gas interface	[21]
Wenzel	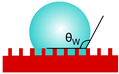	cosθ_W_ = r·cosθ_Y_ r—roughness factor	a liquid droplet remains in contact with peaks and valleys of the rough surface (homogenous wetting)	[22]
Cassie–Baxter	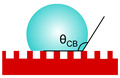	cosθ_CB_ = f_s_·cosθ_Y_ + f_s_ − 1 f_s_—area fraction of solid phase of the rough surface	a liquid droplet is suspended by the surface peaks and does not penetrate the valleys occupied with air (heterogeneous wetting)	[23]
Marmur	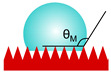	cosθ_M_ = r_f_·f_s_·cosθ_Y_ + f_s_ − 1 r_f_—roughness of the solid that touches the liquid f_s_—area fraction of solid phase of the rough surface	a liquid droplet partially wets the surface and partially sits on the air pockets (heterogeneous wetting)	[24]
Cassie-type states	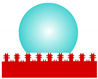 micro-Cassie state	micro-Cassie state: the plastron of microscale dimensions occupies the space between microposts	reversible, localized, and instantaneous transition between two Cassie wetting states facilitated by two-level topography of a superhydrophobic surface	[25]
	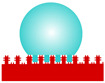 nano-Cassie state	nano-Cassie state: the space between the posts is mostly filled with water, but air remains in the nanofilament layer (hundreds of nanometers thick)		
SLIPS	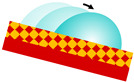	r·(γ_B_·cosθ_B_ − γ_A_·cosθ_A_) − γ_AB_ > 0 r·(γ_B_·cosθ_B_ − γ_A_·cosθ_A_) + γ_A_ − γ_B_ > 0 A—repelled liquid B—lubricating fluid AB—liquid–liquid interface	nano/microstructured porous surface that retains a lubricating fluid, forming stable, smooth, and omniphobic slippery interface	[26]

**Table 2:** Examples of natural superhydrophobic surfaces.

Species	WCA	Surface Structure	Ref.
**Plant Leaves**			
Lotus *Nelumbo nucifiera*	160–162°	papillae covered with dense wax tubules	[31,32,33,34]
Taro *Colocasia esculenta*	150–165°	hierarchical honeycomb-like microstructures	[32,35]
Spurge *Euphorbia myrsinites*	162°	microsized papillae covered with nanosized platelet wax	[32,33]
Rice *Oryza sativa* *	157–162°	papillae (arranged parallely) covered with dense nanopins	[32,34]
Tulip *Tulipa praestans*	160°	convex epidermis covered with dense wax tubules	[32]
Canna *Canna glauca*	159–165°	papillae covered with platelet wax	[32,34]
Nasturtium *Tropaeolum majus*	160°	convex epidermis covered with tiny clumps of waxy tubes	[32]
Ramee *Boehmeria nivea*	164°	unitary structure of micrometer fibers (rear leaf side)	[34]
**Flower Petals**			
Oilseed rape *Brassica campestris* L.	154–155°	interlocking intestine-like microstructures on both petal sides with carotenoid pigment in upper epidermis cells	[36]
Rose *Rosa rubiginosa* **	151–152°	tight-packed periodic arrays of microscale bumps with nanoscale striae (wrinkled folds) on the top of each micropapilla	[37,38]
**Insects**			
Butterfly wing *	151–152°	hierarchical structure composed of overlapping scales with longitudinal ridges and nanoscale grooves	[39,40]
Cicada wing	140–164°	a nanopillar array structure, with regularly spaced, cone-shaped protrusions	[41,42]
Water strider leg *	168–170°	hierarchical needle-like microsetae decorated with nanoscale grooves	[43,44]

* Anisotropic wettability—a liquid droplet rolls off more easily in one direction due to directional variations in the surface structure. ** Adhesive surface of a rose petal can retain droplets by pinning them with densely packed surface structures, though adhesion weakens for higher bump density [51] and for water droplets larger than 10 µL [37].

**Table 5:** Fabrication techniques and properties of superhydrophobic-superoleophobic coatings electrodeposited from different electrolytes.

Amphiphobicity Drivers	Coating	Fabrication Technique	Surface Topography	WCA OCA	Corrosion Resistance *	Ref.
**One-step Electrolysis**						
surface roughness + molecules of low surface energy	Ni	chloride bath; galvanostatic deposition; modification with perfluorooctanoic acid	hierarchical cauliflower-like clusters	160° 152°	−800 mV 10^−6^ A/cm^2^	[109]
	Zn	sulfate bath; galvanostatic deposition; modification with stearic acid; modification with TiO_2_/FAS	hierarchical protrusions	162° 152°	no data	[110]
	Co	sulfate bath; galvanostatic deposition; activation in ethanol–hexane mixture; modification with trichloro perfluorooctyl silane	micro-scaled pyramids with nano-scaled fur-like structures	161° 141°	10^−7^ A/cm^2^ stable properties in air for 6 months	[111]
	Ni-Cu	sulfate bath; galvanostatic deposition; modification with perfluorodecyltrimethoxysilane	pagoda-like micro/nano structures	163° 155°	−250 mV 10^−6^ A/cm^2^	[112]
surface roughness of low surface energy	Ni	chloride–absolute ethanol bath + myristic acid modifier	mushroom-like structures	172° 160°	stable properties after 5 months in air	[113]
	Co	chloride–absolute ethanol bath + myristic acid modifier	sedum-like structures	172° 160°	−200 mV 10^−7^ A/cm^2^	[114]
**Two-step Electrolysis**						
surface roughness + molecules of low surface energy	Zn	sulfate bath; galvanostatic depositions; modification with pentadecafluorooctanoic acid	concave structures on hexagonal crystals	153° 149°	stable properties in air for 120 days	[115]
	Zn	sulfate bath; galvanostatic depositions; modification with perfluorooctanoic acid	hierarchical structure with grooves	155° 154°	stable properties in air for 6 months, in NaCl solution	[116]
	Ni	laser ablation; sulfate bath + ethylenediamine modifier; galvanostatic depositions; modification with perfluorooctanoic acid	hierarchical micro- and nanocones	161° 153°	no data	[117]

* Corrosion potential versus reference electrode (saturated calomel electrode or Ag/AgCl).

**Table 6:** Fabrication techniques and properties of electrodeposited slippery coatings.

Coating	Fabrication Technique	Surface Topography	WCA TA	Corrosion Resistance *	Ref.
Ag	nitrate–ammonia bath; galvanostatic deposition; modification with dodecanethiol; dimethyl silicone oil lubricant	hierarchical dendrites	no data 8°	no data	[119]
Co	chloride-sulfate bath; potentiostatic deposition; chemical oxidation; modification with dopamine; modification with dodecanethiol; dimethyl silicone oil lubricant	hierarchical dendrites	96° 6°	−220 mV 10^−6^ A/cm^2^	[120]
Cu	sulfate bath; two-step potentiostatic deposition; chemical oxidation; modification with stearic acid; silicone oil lubricant	hierarchical cauliflower-like clusters	93° 3°	−220 mV 10^−7^–10^−6^ A/cm^2^	[121]
Cu	sulfate bath; two-step deposition: potentiostatic–galvanostatic; modification with n-hexa methyl mercaptan; perfluoroether oil lubricant	boulder-like, needle-like or cauliflower-like	120° 2° **	stabile properties for 1.5-years	[122]
Ni	chloride bath; two-step galvanostatic deposition, hydrogen-bubble template; modification with myristic acid; paraffin lubricant	hierarchical cauliflower-like clusters	108/40° *** 33/7°	10^5^ Ω∙cm^2^	[123]
Cu	chloride bath; potentiostatic Cu deposition; anodic oxidation; modification with dodecanethiol; perfluorinated lubricant	nanoscale bundle clusters	no data 11°	−220 mV 10^−4^ A/cm^2^	[124]
Fe	sulfate bath; potentiostatic deposition; chemical oxidation; modification with dopamine; modification with dodecanethiol; deep eutectic solvent lubricant	dendritic wire clusters with ravine-like gaps	82° 8°	−260 mV 10^−3^ A/cm^2^	[125]
Cu	sulfate bath; potentiostatic deposition; modification with carbon fibers; perfluorinated lubricant	sponge-like	no data 5°	10^−7^A/cm^2^	[126]
Ni/TiO_2_	Watts bath; constant voltage deposition; modification with myristic acid; perfluorinated lubricant	hierarchical flower-like	118° 4°	200 mV 10^−8^ A/cm^2^	[127]

* Corrosion potential versus reference electrode (saturated calomel electrode or Ag/AgCl). ** Contact angle hysteresis. *** WCA and TA angles for solid/liquid lubricant states.

**Table 7:** Attachment of microorganisms (cells/cm^2^) to bare, superhydrophobic, and slippery surfaces *.

Microorganism	Contact Time	Bare Surface	Slippery Surface	Ref.
Diatom *Navicula minima*	3 days	Ti: 1.4 × 10^10^ Ti/SHC: 4.1 × 10^8^	Ti/SC: 6.6 × 10^6^	[119]
	14 days	Ti: 1.6 × 10^11^ Ti/SHC: 2.5 × 10^9^	Ti/SC: 6.8 × 10^7^	
Green algae *Chlorella vulgaris*	7 days	Ti: 7.7 × 10^10^ Ti/SHC: 5.5 × 10^8^	Ti/SC: 9.9 × 10^6^	
	14 days	Ti: 1.6 × 10^11^ Ti/SHC: 5.6 × 10^9^	Ti/SC: 5.0 × 10^7^	
Sulfate-reducing bacteria culture	3 days	Cu: 1.3 × 10^10^	Cu/SC: 1.3 × 10^9^	[120]
	24 h	CuZn: 3.4 × 10^6^	CuZn/SC: 2.9 × 10^5^	[124]
Diatom *Navicula minima*	14 days	Cu: 2.0 × 10^11^	Cu/SC: 2.0 × 10^7^	[125]
Green algae *Chlorella vulgaris*	14 days	no data	Cu/SC: 3.1 × 10^7^	
Sulfate-reducing bacteria culture	7 days	Cu: 3.0 × 10^10^	Cu/SC: 4.0 × 10^6^	

* Slippery coating (SC) or superhydrophobic coating (SHC) type based on references in Table 6.

## Data Availability

No new data were created or analyzed in this study. Data sharing is not applicable to this article.

## Appendix A

In the context of surface wettability by a single class of liquids, such as water or oil, the terminology used to describe extreme wetting behavior is well defined. Commonly accepted terms include **superhydrophobicity**–**superhydrophilicity** for water, as well as **superoleophobicity**–**superoleophilicity** for oils. However, when surfaces interact with both water and oils or other low-surface-tension liquids, the nomenclature becomes less straightforward. In scientific literature [12,16,17,19,28,30], several additional terms are encountered, including **superamphiphobicity**–**superamphiphilicity**, **superomniphobicity**–**superomniphilicity**, **superhygrophobicity**–**superhygrophilicity**, and **superlyophobicity**–**superlyophilicity**. These terms are derived from Greek or Latin words, such as *hydro* (Greek: water), *oleo* (Latin: oil), *elaio* (Greek: oil), *omnis* (Latin: all), *amphi* (Greek: both or dual nature), *hygro* (Greek: liquid), *lyo* (Greek: solvent or to dissolve), *phobos* (Greek: fear or aversion), and *philos* (Greek: friend or affinity). Accordingly, the terms **superamphiphobicity**–**superamphiphilicity** are used to describe surfaces that repel or are wetted by two classes of liquids: water and oils, the latter typically having surface tensions around 30 mN/m. The terms **superomniphobicity**–**superomniphilicity** refer to surfaces that repel or are fully wetted by all types of liquids, including water with high surface tension (around 73 mN/m), as well as oils and other polar or nonpolar liquids with much lower surface tensions (as low as 10–20 mN/m). Less frequently used terms like **superhygrophobicity**–**superhygrophilicity** and **superlyophobicity**–**superlyophilicity** refer to wetting behavior involving liquids of varying surface tensions. In practice, these terms are often used interchangeably, which can lead to inconsistencies. In each case, the conventional classification of superwetting behavior is based on contact angle measurements. Surfaces are defined as super(…)phobic when the contact angle exceeds 150°, and as super(…)philic when the contact angle is less than 10°.
